# Polylactide—Meso-Substituted Arylporphyrin Composites: Structure, Properties and Antibacterial Activity

**DOI:** 10.3390/polym15041027

**Published:** 2023-02-18

**Authors:** Yulia V. Tertyshnaya, Anton V. Lobanov, Egor S. Morokov, Grigorii A. Buzanov, Zubarzhat R. Abushakhmanova

**Affiliations:** 1 Department of Biological and Chemical Physics of Polymers, Emanuel Institute of Biochemical Physics, Russian Academy of Sciences, 4 Kosygina Str., Moscow 119334, Russia; 2 Laboratory Advanced Composite Materials and Technologies 36 Stremyanniy, Plekhanov Russian University of Economics, Moscow 117997, Russia; 3 Department Physics and Mathematics, Pirogov Russian National Research Medical University, 1 Ostrovityanova Str, Moscow 117997, Russia; 4 Kurnakov Institute of General and Inorganic Chemistry, Russian Academy of Sciences, 31 Leninskiy Pr., Moscow 119991, Russia

**Keywords:** polylactide, arylporphyrin, polymer–porphyrin composites, X-ray diffractogram, degree of crystallinity, bioactivity, DSC

## Abstract

The structural features and antibacterial properties of polymer–porphyrin composites were investigated. Meso-substituted arylporphyrin 0.2–0.5 wt.% was immobilized in a polylactide matrix. The immobilization of porphyrin causes a bathochromic shift and splitting of the Soret band. This study of the morphology of the obtained composites demonstrated a uniform distribution of the meso-substituted arylporphyrin in the polylactide matrix. It was determined by the X-ray diffraction analysis that porphyrin does not affect the α-form of polylactide crystalline formations. However, its addition into the polymer somewhat reduces the melting point (by 1–2 °C) and the degree of crystallinity of polylactide (by 3–4%). The elastic characteristics of the resulting systems were determined by the ultrasonic method, and a decrease in the density of the samples with an increase of the arylporphyrin content was shown. According to the results of the biological test, the dark toxicity of the obtained composites against the microorganisms *Staphylococcus aureus*, *Salmonella* Typhimurium and *Escherichia coli* was shown. Immobilizates containing 0.4 and 0.5 wt.% porphyrin showed the best antibacterial effect. The antibacterial activity of the studied composites makes it possible to attribute the polylactide–porphyrin systems to promising materials in the field of medicine and bioengineering.

## 1. Introduction

Currently, the chemistry of porphyrins and porphyrin polymers is a constantly developing field. Due to the unique molecular structure of tetrapyrrole macroheterocyclic compounds and the possibility of their modification, porphyrins are widely used in medicine, engineering, and biotechnology [1,2,3]. Such an interest is owed to their diverse biological functions as well as photochemical and photophysical properties [4,5,6]. Porphyrins can differ in bridging groups, substituents in the meso-positions (5,10,15,20) of the macroring, and the closure of neighboring pyrrole substituents into rings. Hydrogenation of pyrrole double bonds is also possible. Changes may affect the macroring itself: it may contain heteroatoms, be methylated, and additional rings may be attached to it. All tetrapyrrole macroheterocyclic compounds introduced into the polymer should be divided into two large groups of polymer–porphyrin systems:

- Physically fixed porphyrins;
- Chemically bound porphyrins.

The group of physically fixed porphyrins consists of systems in which the porphyrin is bound to the carrier mainly by nonspecific sorption forces and can be fixed either on the surface of the carrier or be included inside the polymer shell. Binding between porphyrins or their analogues is carried out through a comonomer or a linking bridge.

There are several ways to physically immobilize porphyrin and obtain composites: dispersion in a polymer solution; joint dissolution of the polymer and porphyrin, followed by film casting; and impregnation of the finished polymer carrier with a porphyrin solution [7,8,9].

Chemically immobilized porphyrins represent another numerous subgroup of porphyrin polymers [10,11,12]. They contain a tetrapyrrole macrocycle in the side chain of the polymer that is attached to the *β*- or meso-position using a spacer of various lengths.

Due to the combination of tetrapyrrole macroheterocycles and macromolecular compounds, great opportunities open up for the synthesis of molecular structures characterized by specific properties that are easy to control and change in accordance with their field of application [13,14,15].

In previous research [16], star-shaped block copolymers of poly(L-lactide)-b-poly(ethylene glycol) (PLA-b-PEG) with porphyrin cores were synthesized. The PLA-b-PEG copolymer exhibits a high yield of singlet oxygen, when exposed to visible light and high fluorescence quantum yields and can be used for delivery systems for chemotherapeutic drugs. In another study [17], polymer nanoparticles with gold into which porphyrin was introduced to improve their photosensitivity were studied in order to use this copolymer for the treatment of cancer and the production of antibacterial drugs. It was determined that Au nanoparticles were more stable and uniformly distributed on the surface of the copolymer, and polymer–Au nanoparticles obtained in the form of a core–shell can be a suitable platform for the development of effective delivery systems in combination with photodynamic and photothermal therapy of cancer cells due to their high production of singlet oxygen and heat. In other research [18], a material based on PLA and an iron(III) complex with tetraphenylporphyrin (FeClTPP) was obtained and studied. While studying the composite material PLA–FeClTPP, the ability of the porphyrin metal complex to influence the thermophysical characteristics and strength properties of composites was found. It was shown that the presence of FeClTPP reduces the melting point of PLA matrix by 1–3 °C and the degree of its crystallinity by 3−4% but does not adversely influence the mechanical characteristics of the polymer matrix.

In an experiment [19], a bromo derivative of zinc metalloporphyrin and its immobilization in polyvinyl alcohol (PVA) were considered. The antibacterial activity of the obtained systems against *E. coli* (*Escherichia coli*), fungal infection (*Candida albicans*), and *Staphylococcus aureus* was studied. Along with a relatively even effect of all porphyrin-polymers on *Staphylococcus aureus* and E.coli, a high rate of growth of the *Candida albicans* culture was observed when using nonmetal bromine-containing immobilizates. The immobilizate containing zinc-2-formyl-10,15-(4′,4′-dibromophenyl)-5,20-diphenylporphyrine had a significant effect on the inhibition of given cultures.

Since there is a problem of spreading infections and resistance of microorganisms to widely used drugs, the creation and study of new functional bioactive materials is very important. Supramolecular polymer–porphyrin systems can expand the scope of both biodegradable polymers and porphyrins. It should be noted that the current work is a continuation of another study [7]. A metal-free meso-arylporphyrin immobilizate with alkyl side substituents is now being studied. In this work, a supramolecular porphyrin-polymer system based on polylactide and 5,10,15,20-tetrakis(4-*n*-hexyloxyphenyl)porphyrin was obtained using physical immobilization. Physical immobilization is a less complex and time-consuming method compared to chemical immobilization. It is assumed that in this way, it is possible to create porphyrin–polymer composites based on a biodegradable polymer and porphyrin and the resulting materials will exhibit antibacterial activity.

Polylactide is a well-known linear polyester obtained from renewable plant resources [20,21,22]. It is one of the biodegradable polymers that can be used as a promising alternative to petroleum-based polymers. PLA has good thermoplasticity, tensile strength, and a high degree of transparency. It is biocompatible with a human body, undergoes biodegradation, and does not form toxic waste, which is why PLA is actively studied investigated and used in various fields: packaging, the food industry, tissue engineering, biotechnology, and medicine [23,24,25].

The purpose of this research is to obtain a supramolecular polymer–porphyrin system; to study the mutual influence of the components during porphyrin immobilization in a polylactide matrix using thermophysical, spectral, and optical methods; and to evaluate the possibility of using the obtained composites as bioactive materials by conducting biological testing.

## 2. Materials and Methods

### 2.1. Sample Preparation

**Figure 1:**
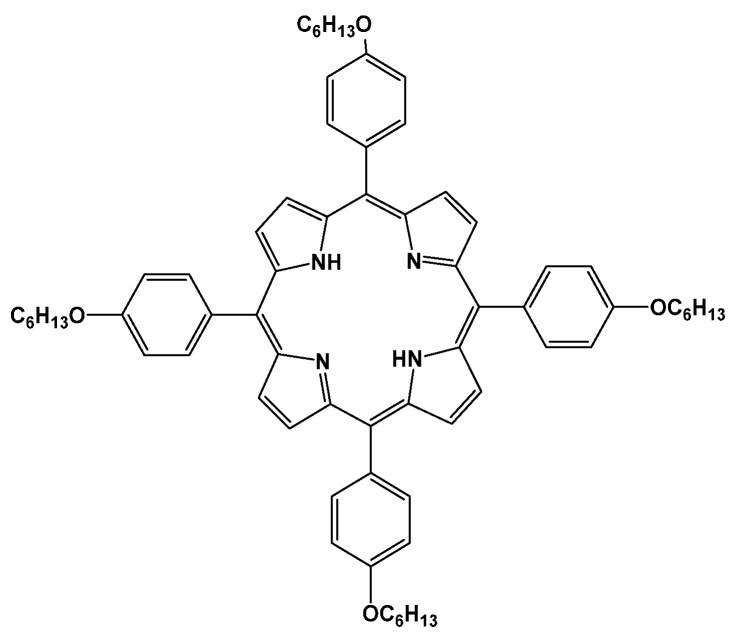
Molecular structure of 5,10,15,20-tetrakis(4-*n*-hexyloxyphenyl)porphyrin.

Thermoplastic PLA—poly(lactic acid), 4032D (with about 2% of D-lactide) with molecular weight (M_w_) of 1.7 × 10^5^ g/mol was procured from Nature Works (Minnetonka, MN, USA). Low molecular weight component (Figure 1) 5,10,15,20-tetrakis(4-*n*-hexyloxyphenyl)porphyrin (TPP4OC_6_) synthesized as described in [26].

**Scheme 1:**
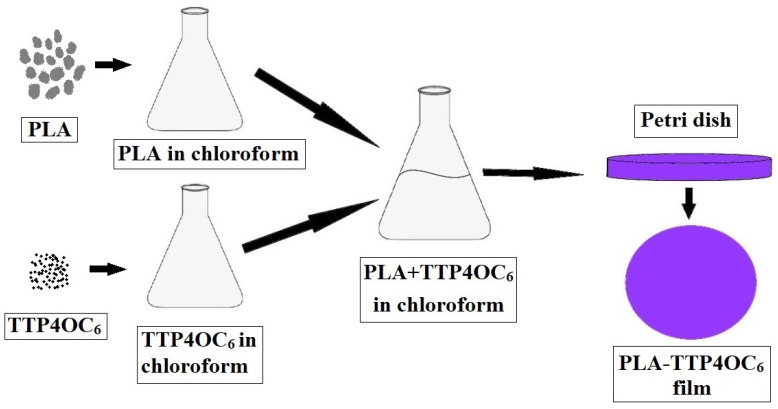
PLA–TPP4OC_6_ composite production.

Film samples of PLA–TPP4OC_6_ with a diameter of 90 mm and a thickness of 100–120 µm were obtained by casting from a solution (Scheme 1). The solvent was chloroform (Vekton, Russia). PLA was dissolved in chloroform and heated to a temperature of 59 ± 1 °C. After cooling the PLA solution to 35–38 °C, the porphyrin solution was added. The resulting solution was mixed and poured into Petri dishes. The content of TPP4OC_6_ porphyrins in the film composites was 0.2–0.5 wt %.

### 2.2. Analysis of Crystallization

The temperature characteristics and crystallinity degree of the samples were determined by the DSC method using a differential scanning calorimeter DSC 204 F1 (Netzsch, Germany) under a nitrogen atmosphere. Samples of about 5.0–5.5 mg sealed in aluminum pans were heated from 20 to 200 °C at a rate of 10 °C/min. Calibration was carried out with Indium (T_m_ = 156.6 °C). The crystallinity degree of PLA (χ_c_) was calculated as

χ_c_ (%) = (Δ*H*_m_/Δ*H*_m_*) × 100%,

where Δ*H*_m_ is the melting enthalpy (an experiment result), Δ*H*_m_* is the enthalpy assuming 100% crystalline PLA homopolymer 93.1 J/g [27].

### 2.3. Morphology of the Sample

The morphology of the pure PLA and PLA–TPP4OC_6_ samples was characterized using optical microscopy with a Leica LMDM (Germany, Wetzlar).

### 2.4. X-ray Analysis

X-ray diffraction studies were performed at the Centre of Shared Equipment of IGIC RAS using Bruker D8 Advance (Billerica, MA, USA) diffractometer (CuKα) radiation, Ni-filter, reflection geometry, LYNXEYE detector) in the 2θ range of 5–60° and 0.01125° step and exposition time of 0.15–0.25 s per step. The film samples under study were placed on a planar low-background single crystal silicon cuvettes.

### 2.5. UV-vis Spectroscopyy

UV-vis absorption spectra of the film samples were recorded between 300 and 800 nm wavelength using a DR/4000V spectrophotometer of HACH-Lange (Loveland, Colorado, USA). To record the spectrum, a piece of a film polymer composite 2 × 2 cm^2^ in size was cut out and fixed in the cuvette section of the spectrophotometer in a plane perpendicular to the direction of the light flux.

### 2.6. Ultrasonic Tests

The evaluation of the elastic characteristics of polymer samples was carried out using a SIAM 2017 pulsed acoustic microscope designed and built at the Institute of Biochemical Physics of the Russian Academy of Sciences. A detailed description of the structure of the microscope and the principles of visualization and calculation of elastic properties are described in [28]. The calculation of elastic characteristics was carried out based on the analysis of echograms, which were used to measure the propagation velocities of longitudinal *C_L_* and transverse sound waves *C_T_.* Together with the data on the density of the material *ρ*, the elastic moduli were calculated—the shear modulus *G*, the modulus of all-round compression *K*, the modulus of elasticity *E*, and Poisson’s ratio *µ*:

(1) $$G=C_{T}^{2}\cdot \rho$$

(2) $$K=C_{L}^{2}\cdot \rho -\frac{4}{3}\cdot C_{T}^{2}\cdot \rho$$

(3) $$E=\frac{9\cdot K\cdot G}{3K+G}$$

(4) $$\mu =\frac{E}{2G}-1$$

### 2.7. Bacteriological Test

Film samples were subjected to bioassays on cellular material. *S. aureus* p 209 (*Staphylococcus aureus*), *S.* Typhimurium (*Salmonella* Typhimurium), *E. coli* 1257 (*E. coli*) were used as test cultures. The cultures of microorganisms were kindly supplied by the All-Russian Research Institute for Veterinary Sanitation, Hygiene, and Ecology (Moscow, Russia). The test microorganism cultures were inoculated onto meat peptone agar and incubated for 23 h at 37 °C. Then, a suspension of each microorganism was prepared in physiological saline and the concentration of microbial cells was determined according to the turbidity standard of 104 CFU/mL. A film sample, with an area of 1 × 1 cm^2^, was placed in sterile Petri dishes, to which 1 mL of the test culture suspension was added and kept at room temperature for 60 min. Then, 10 mL of sterile saline solution was poured into the dish and kept for 15 min to elute the test culture from the suspension particles. After exposure, the material from the dishes in the amount of 100 µL was sown on the surface of meat peptone agar, previously poured into Petri dishes. The inoculations were incubated for 48 h at 37 °C. At the same time, the suspensions of test cultures used in the experiment were inoculated to control the concentration of viable microorganisms. Then, the number of colonies of viable microorganisms grown on the surface of the agar was counted.

### 2.8. Statistical Processing

The experimental data obtained in the work was calculated as the arithmetic mean and its standard error. The calculations were executed using Statistica 8.0 software (Dell Software Inc., Round Rock, TX, USA) and Microsoft Excel 2007. The significant differences were evaluated using Student’s *t*-test (* represents *p* < 0.001, ** represents *p* < 0.05).

## 3. Results and Discussion

### 3.1. Morphology

**Figure 2:**
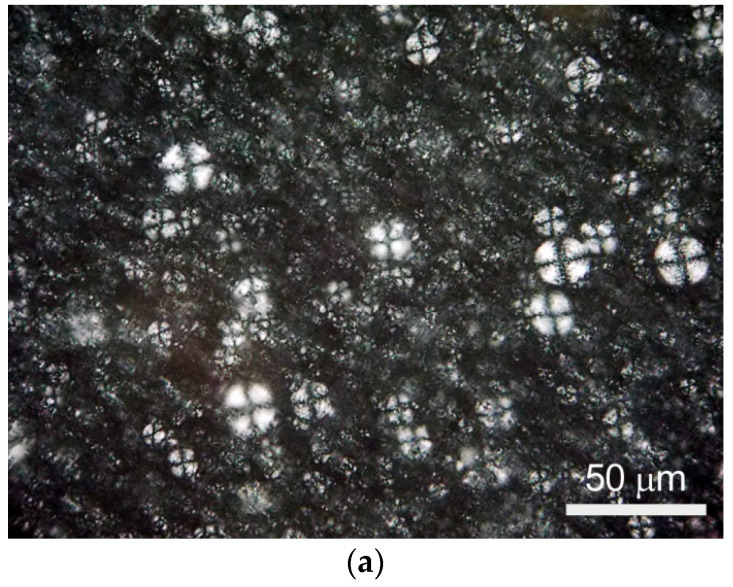

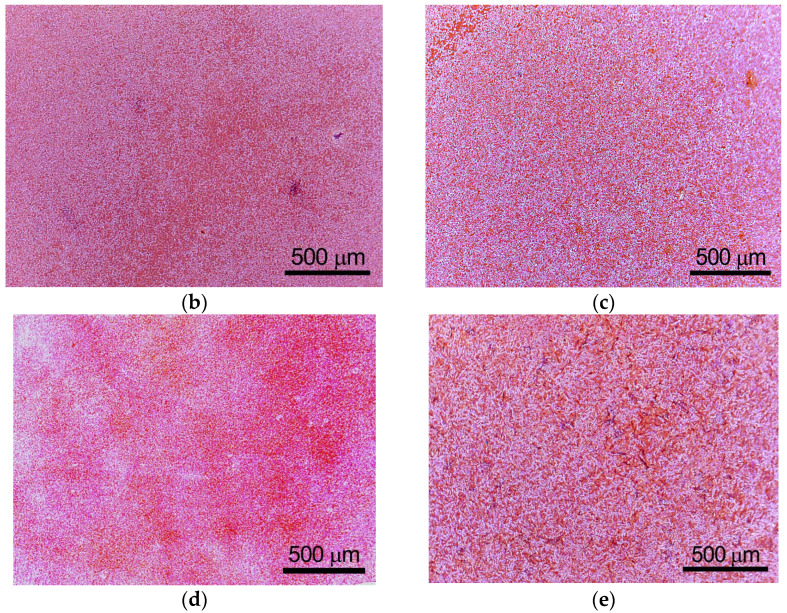
Optical micrographs of PLA (**a**) and composites PLA-TPP4OC_6_ with TPP4OC_6_ content, wt.%: 0.2 (**b**), 0.3 (**c**), 0.4 (**d**), and 0.5 (**e**).

First, the morphology of the PLA–TPP4OC_6_ samples was studied. Micrographs of PLA and polymer–porphyrin composites were obtained by optical microscopy. The polylactide obtained from a chloroform solution has a spherulitic structure (Figure 2a). Figure 2b–e shows the images of the samples in which meso-substituted porphyrin is present in the PLA matrix as submicroinclusions. According to Figure 2b–e, the addition of TPP4OC_6_ in an amount of 0.2–0.4 wt.% has a similar distribution pattern in the polymer matrix.

TPP4OC_6_ submicroinclusions are evenly distributed. With a TPP4OC_6_ content of 0.5 wt. % in the PLA matrix (Figure 2e), the micrograph differs from the previous ones, which is apparently due to the tendency of extended alkyl substituents of 5,10,15,20-tetrakis(4-*n*-hexyloxyphenyl) porphyrin to aggregate at certain concentrations. It is interesting and important to study the influence of the content of immobilized porphyrin on the structure and properties of the polymer–porphyrin composites.

### 3.2. X-ray Diffraction

**Figure 3:**
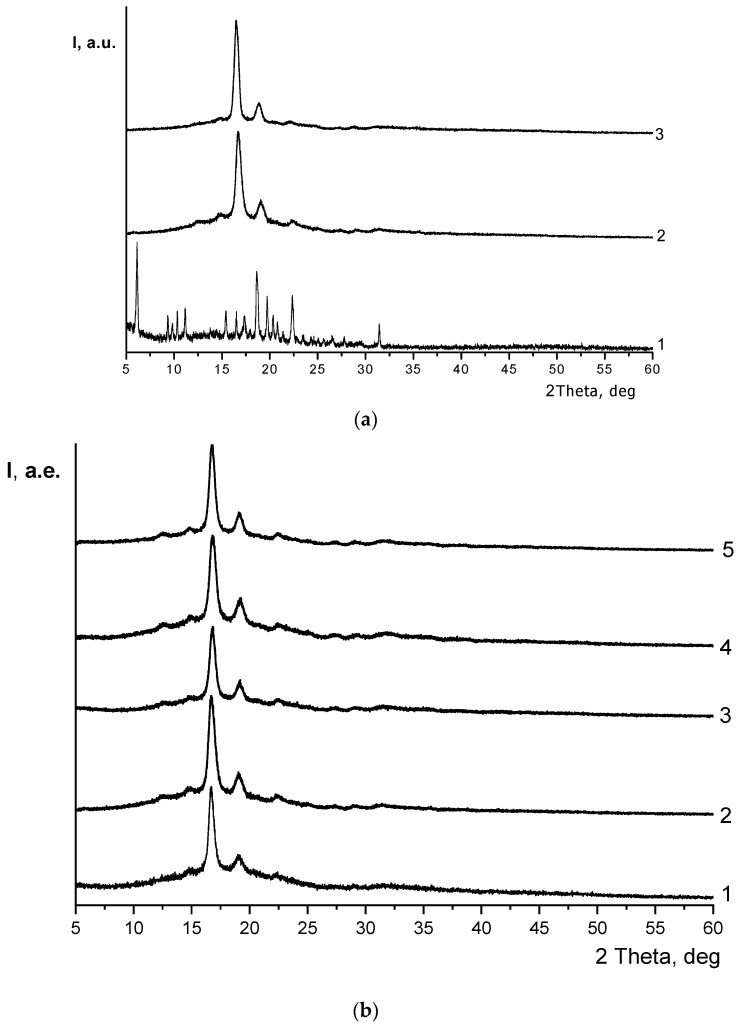
(**a**) Diffractograms of TPP4OC_6_ (1), PLA granules (2), and PLA film (3). (**b**) Diffractograms PLA (1) and composites PLA–TPP4OC_6_ with TPP4OC_6_ content, wt.%: 0.2 (2), 0.3 (3), 0.4 (4), and 0.5 (5).

X-ray diffraction analysis (XRD) was used to receive diffraction patterns of the initial substances: PLA granules, TPP4OC_6_ crystals, and a PLA film sample obtained from a solution (Figure 3a).

It was determined that the diffraction patterns of the PLA granules and the film sample were identical. The study of all compositions showed that different content of 5,10,15,20-tetrakis(4-*n*-hexyloxyphenyl)porphyrin immobilized in the PLA matrix did not affect the modification of polylactide crystalline formations. In this experiment, the diffraction patterns of pure PLA and all PLA–TPP4OC_6_ samples are identical (Figure 3b). One can see a narrow intense and two weak diffraction peaks on the left and right sides, the position of which on the abscissa axis corresponds to the position of the main reflections of the α-form—the orthorhombic lattice of polylactide: 2θ = 12.5, 14.8, 16.8, 19.1, 22.5° [29,30,31].

### 3.3. Differential Scanning Calorimetry (DSC)

**Figure 4:**
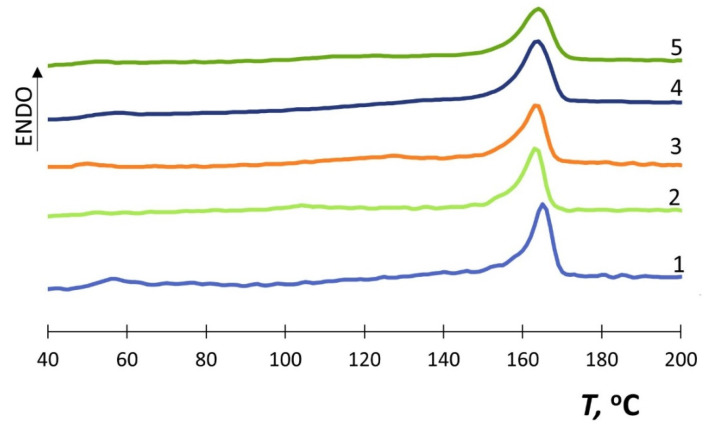
DSC thermograms of PLA (1) and composites PLA–TPP4OC_6_ with TPP4OC_6_ content, wt.%: 0.2 (2), 0.3 (3), 0.4 (4), and 0.5 (5).

The study of the crystal structure was continued by the thermal method. The DSC method was used to determine the thermophysical characteristics of the obtained samples of polymer–porphyrin composites. The type of thermograms is shown in Figure 4. It was found that the sample preparation method had significantly affected the self-organization of the PLA–TPP4OC_6_ compositions. It seems that heating the PLA solution leads to the disappearance of the cold crystallization peak (Figure 4), which is characteristic of this polymer [32,33,34]. The peak of cold crystallization is not observed due to the completion of the crystallization process when the film is formed from the solution. Then, when heated, recrystallization does not occur and there is no exothermic peak of cold crystallization.

**Figure 5:**
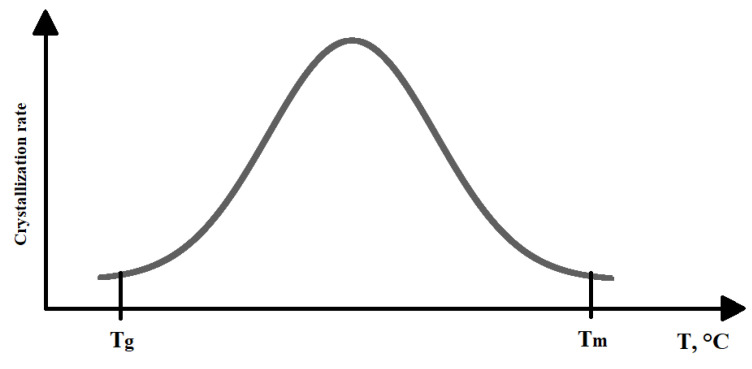
Temperature dependence of the crystallization rate (a theoretical curve).

Apparently, when the PLA solution in chloroform is heated above the PLA glass transition temperature, the segmental mobility of macromolecules increases. Furthermore, when the solution is cooled in Petri dishes and the solvent gradually evaporates, the polylactide chains have time to fit into domains and form a complete crystalline structure—spherulites—because the crystallization process is accelerated. The dependence of the crystallization rate on temperature is expressed by a curve with a maximum (Figure 5).

Consequently, at a temperature above T_g_, but below T_m_, segmental mobility of the polymer increases, and favorable conditions are created for rapid nucleation and rapid growth of crystalline formations. Under such conditions, even in the non-isothermal regime of PLA crystallization, there is enough time to form a crystal structure without subsequent precrystallization.

In turn, the addition of a low molecular weight component, 5,10,15,20-tetrakis(4-n-hexyloxyphenyl)porphyrin, leads to a weakly pronounced glass transition process, the temperature of which is difficult to determine (the data in Table 1 are provided in parentheses).

The presence of a low-molecular weight substance in the polymer matrix can cause a dilution effect, thereby reducing the value of T_g_ and expanding the interval in which the polymer retains its deformability. The melting temperature of PLA in composites varies slightly by 1–2 °C but tends to decrease compared to 100% PLA. A similar dependence is demonstrated by the degree of PLA crystallinity.

A slight decrease in the values of χ_c_ in PLA–TPP composites was observed in [18] at low concentrations of the porphyrin metal complex (0.013–0.132 wt. %). A similar effect was shown by the authors in the work [35] at a high content of tetraphenylporphyrin in the polymer matrix. The decrease in the degree of crystallinity may be associated with a change in the intermolecular interaction in polylactide due to the addition of a low molecular-weight substance.

### 3.4. UV-vis Spectra

**Figure 6:**
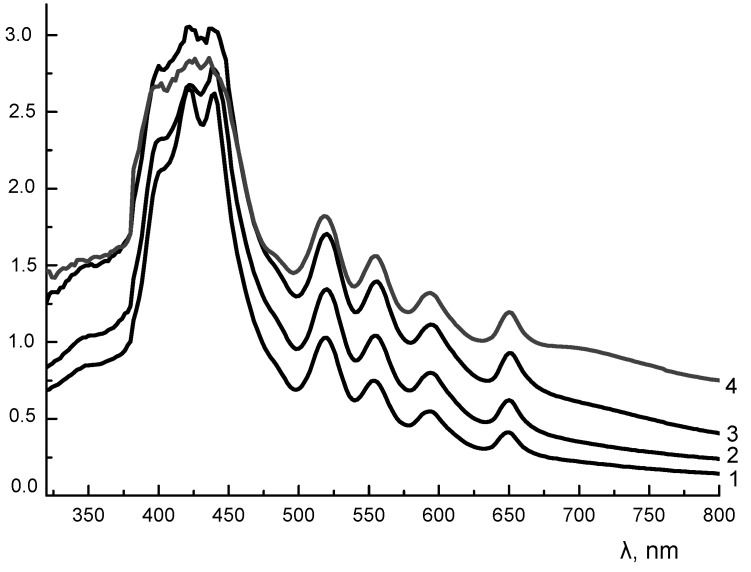
UV-vis spectra of PLA–TPP4OC_6_ composites with TPP4OC_6_ content, wt.%: 0.2 (1), 0.3 (2), 0.4 (3), and 0.5 (4).

However, not only porphyrin affects PLA, but also polylactide affects TPP4OC_6_ during immobilization of the latter. Electronic absorption spectra of porphyrin were obtained for PLA–TPP4OC_6_ film samples. It should be noted that the UV-vis spectrum of 5,10,15,20-tetrakis(4-*n*-hexyloxyphenyl)porphyrin in a chloroform solution contains five bands: an intense Soret band at 420 nm and four less intense Q-bands [26]. The studied compound belongs to the ethio-type with the intensity of Q-bands εI > εII > εIII > εIV, characteristic of symmetrically substituted porphyrins [36]. The presence of porphyrin in the PLA matrix and the nature of the distribution of the substituted porphyrin TPP4OC_6_ are shown in Figure 6.

Regardless of the content of TPP4OC_6_ in the PLA matrix, the ethio-type is preserved in the UV-vis spectra for all studied composites.

The presence of the Soret band is a characteristic feature of all tetrapyrrole macrocycles. The Soret band of 5,10,15,20-tetrakis(4-*n*-hexyloxyphenyl)porphyrin immobilized in the PLA matrix splits showing a bathochromic shift and forming bands at 425 and 447 nm. The bathochromic shift (“red” shift) is observed for many organic molecules when the polarity of their ambience changes [37]. For example, a bathochromic shift is observed for dyes, in particular, upon solubilization into micelles [38]. The immobilization of TPP4OC_6_ in polylactide whose macrochains contain carboxyl groups and an ether bond, most likely affects the electron density distribution relative to the porphyrin and provokes a shift of the absorption maximum to a long wavelength region. In addition, the spectra of all composites show a shoulder at 400 nm (“blue” shift) while at the same time the Q-bands (II, III and IV) are slightly shifted to higher wavelengths. These phenomena are related to the formation of H-type aggregates, which is a consequence of the porphyrin immobilization in the PLA matrix [39,40,41].

Thus, having studied the structural characteristics, we would like to evaluate the effect of TPP4OC_6_ on the properties of the obtained supramolecular systems. For these purposes, ultrasonic measurements and a microbiological test were used.

### 3.5. Ultrasonic Measurements

Ultrasonic data were used to calculate the elastic moduli of polymer–porphyrin compositions. Results are presented in Table 2.

The addition of a low-molecular component to the volume of PLA significantly affects both the density of the material, the value of which decreases to 1.24–1.23 g/cm^3^, and the propagation velocity of shear elastic waves, the value of which drops to 1.12 km/s. The P-wave propagation velocity is insensitive to low porphyrin concentrations and is 2.28 km/s. An increase in the concentration of a low molecular weight filler in the polymer leads to a gradual decrease in the elasticity of the material; the decrease in the elastic modulus *E* in a sample with 0.5 wt.% of TPP4OC_6_ content relative to the initial polymer is about 7%. Compression modulus *K* is not particularly sensitive to the presence of TPP4OC_6_ in the PLA matrix. The Poisson ratio of PLA is 0.33; for all samples of PLA–TPP4OC_6_ it is 0.34, which is consistent with the literature data. Most thermoplastics have a Poisson’s ratio in the range of 0.3–0.4, and for elastic materials, such as rubbers, *μ* can be maximum and reach 0.5 [42].

Ultrasonic data correlate with DSC results. The decrease in the density of the PLA-TPP4OC_6_ composites is accompanied by a decrease in the degree of PLA crystallinity and confirms the effect of arylporphyrin on the structure and properties of the polymer matrix.

### 3.6. Biological Tests

The final stage of this work is devoted to the study of antibacterial activity. Due to porphyrins, polymer–porphyrin composites can exhibit dark toxicity, which makes them promising materials outside of photodynamic therapy [19,43].

**Figure 7:**
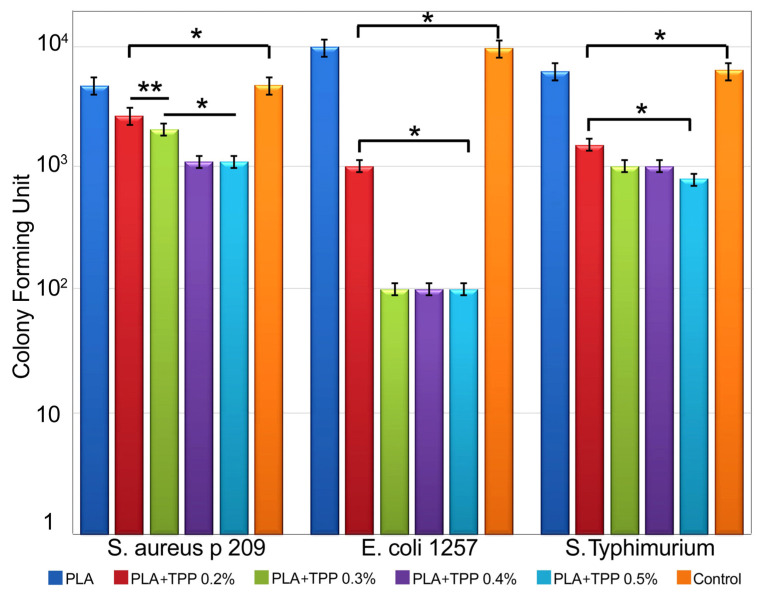
Biological activity of PLA and PLA–TPP4OC_6_ composites. The data are shown as the mean ± SD (n = 5). * represents significant differences *p* < 0.001, and ** represents *p* < 0.05.

Film samples were subjected to bioassays. *S. aureus* p 209 (*Staphylococcus aureus*), *S.* Typhimurium (*Salmonella*), *E. coli* 1257 (*E. coli*) were used as test cultures. The results of the biological tests of PLA–TPP4OC_6_ film samples are shown in Figure 7. It can be seen that the number of viable bacteria of Staphylococcus aureus and Salmonella significantly decreased by 5–7 times in samples containing more than 0.3 wt.% TPP4OC_6_. The high biological activity of the obtained composites is observed in relation to Escherichia coli. The number of viable microorganisms on a sample containing 0.2 wt.% TPP4OC_6_ decreases by 10 times compared to the control, and for samples with a porphyrin content of more than 0.2 wt.%, by 100 times. For the PLA sample, the data practically did not differ from the control measurements. The dark antibacterial activity of polymer–porphyrin systems against *S. aureus* and *E. coli* was also observed by other authors [19].

It can be assumed that the antibacterial properties of porphyrins in the film composites are associated primarily with their effect on the cell walls of microorganisms, by changing their structural properties [44]. Apparently, the porphyrin molecules in the film composite are capable of disturbing the hydrophilic–lipophilic balance of the cell wall of the contacting microorganism.

The effect is observed both in relation to Gram-positive bacteria and Gram-negative ones, which are protected by a second cell membrane. It contains a large number of macromolecules of different chemical types. Gram-negative bacteria are distinguished by such an essential component of their cell walls as the lipopolysaccharide layer [45,46]. Porphyrin molecules, due to their inherent hydrophobicity, tend to interact with lipid fragments, while four nitrogen atoms (of which two atoms are amine nitrogen and two other atoms are imine nitrogen) allow porphyrins to form hydrogen bonds with sugar residues. Thus, the features of the chemical structure of the cell wall, apparently, provide improved binding of porphyrins to bacterial cell membranes. As a consequence, porphyrins inhibit the efficiency of adhesion and colonization of pathogens. In particular, this effect is pronounced for samples containing 0.4 and 0.5 wt % porphyrin.

It is known that synthetic compounds containing tetrapyrrol macrocycles are widely used to produce drugs, the therapeutic effect of which is due to activation in response to the effect of an additional factor—light. The bioactivity of porphyrins is initiated by light sources of a certain wavelength (usually, a laser is used); therefore, porphyrins and their complexes with metals are used in photodynamic therapy (PDT) [47,48]. A previous review [47] covers many issues related to the use of photosensitizers for PDT and considers the use of IR-irradiation and laser light to activate the systems under study. The authors [48] investigated star-shaped porphyrin-cored poly(l-lactide)-b-glycopolymer block copolymers. Upon irradiation, copolymers exhibit efficient generation of singlet oxygen and indicate high fluorescence quantum yields; therefore, they can be used for delivery systems of chemotherapeutic drugs.

The resulting PLA–TPP4OC_6_ composites have a certain dark bioactivity, so their use for PDT is ambiguous. Since the efficiency of dark toxicity exceeds the efficiency of phototoxicity, these composites are of little use for PDT. Any other mechanism is highly likely to lead to microbial resistance. This question remains open, and the authors are going to study it further.

## 4. Conclusions

Polymer–porphyrin composites with different contents of meso-arylporphyrin TPP4OC_6_ were obtained using the solution method. The study of the sample morphology by optical microscopy showed a fairly uniform distribution of porphyrin in the polylactide matrix. Study of the PLA–TPP4OC_6_ composites structure made it possible to conclude that the polylactide matrix and meso-arylporphyrin have a mutual influence.

Thus, according to DSC data, the addition of TPP4OC_6_ into the polymer leads to a decrease in the glass transition temperature, which becomes difficult to determine, the melting point (by 1–2 °C) and the degree of crystallinity of polylactide (by 3–4%). However, the α-form of the crystalline formations of polylactide is retained in the PLA–TPP4OC_6_ composites. In turn, the immobilization of porphyrin in the polylactide matrix causes a bathochromic shift and splitting of the Soret band while the ethio-type of the spectrum is retained.

The elastic characteristics of the obtained samples were calculated according to the ultrasonic test. One can see a decrease in sample density with an increase in the content of arylporphyrin from 1.26 g/cm^3^ for PLA to 1.23 g/cm^3^ for a composite with a TPP4OC_6_ content of 0.5 wt.%. The elastic modulus decreases by 5–7% and the shear modulus in composites decreases by 10–12% compared to 100% PLA. The Poisson’s ratio of all composites is 0.34, and it differs a little from the value of μ of pure PLA (0.33). According to the results of the biological test, the dark bioactivity of the obtained composites against the microorganisms *Staphylococcus aureus*, *Salmonella* Typhimurium and *Escherichia coli* was shown. The most pronounced effect was observed in samples containing 0.4 and 0.5 wt.% porphyrin, which allows for the classification of the studied polymer–porphyrin systems as promising materials for bioengineering and medicine.

## 5. Patents

Tertyshnaya Y.V., Zhdanova K.A., Zakharov M.S., Bragina N.A. Biodegradable composite material with antibacterial effect, RU 2752860 C1, 2021, bul. 23.

## Figures and Tables

**Table 1:** Thermophysical characteristics of PLA-ТРР4ОС6 composites.

ТРР4ОС_6_, wt.%	Thermophysical Characteristics			
	T_g_, °C (Δ ± 0.5 °C)	T_m_, °C (Δ ± 0.5 °C)	T_cc_, °C (Δ ± 0.5%)	χ_c_, % (Δ ± 1%)
0	57.5	165	-	37
0.2	(53)	163	-	33
0.3	(53)	163	-	33
0.4	(53)	163	-	34
0.5	(52)	164	-	34

**Table 2:** Results of ultrasonic measurements of the elastic characteristics of PLA–TPP4OC6 composites.

TPP4OC_6_, wt.%	*C_L_*, km/s	*C_T_*, km/s	*ρ*, g/cm^3^	*G*, GPa	*K*, GPa	*E*, GPa	*μ*
0	2.28	1.15	1.26	1.7	4.3	4.4	0.33
0.2	2.28	1.13	1.24	1.6	4.3	4.2	0.34
0.3	2.28	1.12	1.24	1.6	4.4	4.2	0.34
0.4	2.28	1.12	1.24	1.6	4.4	4.2	0.34
0.5	2.28	1.12	1.23	1.5	4.3	4.1	0.34

## Data Availability

The data presented in this study are available on request from the corresponding author.
